# Targeting the PI3K/AKT/mTOR signaling pathway as an effectively radiosensitizing strategy for treating human oral squamous cell carcinoma *in vitro* and *in vivo*

**DOI:** 10.18632/oncotarget.19817

**Published:** 2017-08-02

**Authors:** Chih-Chia Yu, Shih-Kai Hung, Hon-Yi Lin, Wen-Yen Chiou, Moon-Sing Lee, Hui-Fen Liao, Hsien-Bin Huang, Hsu-Chueh Ho, Yu-Chieh Su

**Affiliations:** ^1^ Department of Life Science and Institute of Molecular Biology, National Chung Cheng University, Chia-Yi, Taiwan, R.O.C; ^2^ Department of Radiation Oncology, Dalin Tzu Chi Hospital, Buddhist Tzu Chi Medical Foundation, Taiwan, R.O.C; ^3^ School of Medicine, Tzu Chi University, Hualian, Taiwan, R.O.C; ^4^ Department of Biochemical Science and Technology, National Chiayi University, Chia-Yi, Taiwan, R.O.C; ^5^ Department of Otolaryngology, Dalin Tzu Chi Hospital, Buddhist Tzu Chi Medical Foundation, Taiwan, R.O.C; ^6^ Division of Hematology and Oncology, E-Da Hospital, Kaohsiung, Taiwan; ^7^ School of Medicine, I-Shou University, Kaohsiung, Taiwan, R.O.C

**Keywords:** PI3K/mTOR pathway, radiation, radiosensitization, radioresistant, oral cancer

## Abstract

Radiation therapy (RT) is the current standard adjuvant approach for oral squamous cell carcinoma (OSCC) patients. Radioresistance is a major contributor to radiotherapy failure. In this study, we used patient-derived cells and a radiation-resistant cell line *in vitro* and *in vivo* for two purposes: evaluate the anti-tumor effects and understand the mechanisms in the dual PI3K/mTOR signaling pathway regulation of radiosensitization. Our findings indicate that in OML1-R cells, the radioresistance phenotype is associated with activation of the PI3K/AKT/mTOR signaling pathway. Compared to a combination of PI3K or mTOR inhibitors and radiation, dual blockade of the PI3K and mTOR kinases significantly improved radiation efficacy in oral cancer and patient-derived OSCC cells. Dual PI3K/mTOR inhibition enhanced the effect of radiation by inhibiting AKT/mTOR signaling pathways and caused G1 phase arrest, which is associated with downregulation of cyclin D1/CDK4 activity, leading to growth inhibition. In nude mice xenografted with radioresistant OML1-R cells, the combined treatment was also more effective than RT alone in reducing tumor growth. This treatment was also demonstrated to be dependent on the inhibition of protein kinase-dependent S6 kinase pathway and eIF4E-mediated cap-dependent translation. These findings indicate that activation of the PI3K/AKT/mTOR signaling pathway has a role in radioresistance of OSCC. We determined that a PI3K/mTOR inhibitor combined with radiation exhibits synergistic inhibition of the AKT/mTOR axis and induces cell cycle arrest. Our results show the therapeutic potential of drugs targeting the PI3K/AKT/mTOR signaling pathway should be new candidate drugs for radiosensitization in radiotherapy.

## INTRODUCTION

In Taiwan, oral cancer is the fourth leading cause of cancer death for males. Radiation therapy (RT) is an important and potential treatment modality for oral squamous cell carcinoma (OSCC). However, tumor radioresistance is a major contributor to radiotherapy failure. Although cisplatin and 5-flurouracil (5-FU) are common potent radiosensitizers and combined RT could improve the outcome in locoregionally advanced OSCC [1–3], clinical outcomes clearly show that these advanced approaches cannot completely overcome radioresistance in OSCC. Moreover, toxicities of these drugs not only decrease patients’ quality of life, but also limit overall treatment response. Thus, identifying novel strategies for sensitizing OSCC cells to the effects of ionizing radiation (IR) will improve potency or decrease the toxicity in these patients.

The PI3K/AKT/mTOR signaling pathway has been extensively studied owing to growing evidence supporting its critical role in cancer progression; it influences metabolism, tumor growth, survival, and the development of metastases [4]. Recently, therapeutic compounds that target this pathway have been developed and are currently being evaluated in clinical trials for several malignancies [5] including OSCC cancers [6]. The PI3K/AKT/mTOR signaling pathway is also known to be overstimulated in cancer cells or in tumors resistant to treatments such as chemotherapy, radiation, as well as hormone therapy [3]. Recent findings suggest that dual targeting of PI3K and mTOR has the ability to overcome resistance to IR and improve the anticancer efficacy of the treatment in various cancer cell types *in vitro* and in *in vivo* xenograft models of cancer [7–9].

Although radioresensitizing using dual PI3K/mTOR inhibitors has been reported in head and neck cancer cell lines [10], these results only reflect the OSCC tumor subtype and do not truly represent the clinical situation. In the present study, we used a human radioresistant OSCC cell line that was established by Hon-Yi Lin and Michael W.Y. Chan et al. [11] to examine the radiosensitizing effects of single and dual PI3K/AKT/mTOR inhibitors by using both *in vitro* and *in vivo* models. Particularly, we established a patient-derived OSCC cell culture isolation of tumor tissues that served as a preclinical model. This model could be of clinical value and provide insights into the effects of combination treatments with PI3K/mTOR inhibitors and RT, as well as the putative mechanisms through which they act. Furthermore, our findings showed that dual a PI3K/mTOR inhibitor could efficiently overcome radioresistance in oral cells and sensitized oral carcinoma cells to IR. Therefore, this is a promising therapeutic approach that may replace cisplatin, a drug with known considerable toxicity, to improve treatment results.

## RESULTS

### Inhibition of the PI3K/AKT/mTOR signaling pathway sensitizes radioresistant cells to IR

**Figure 1:**
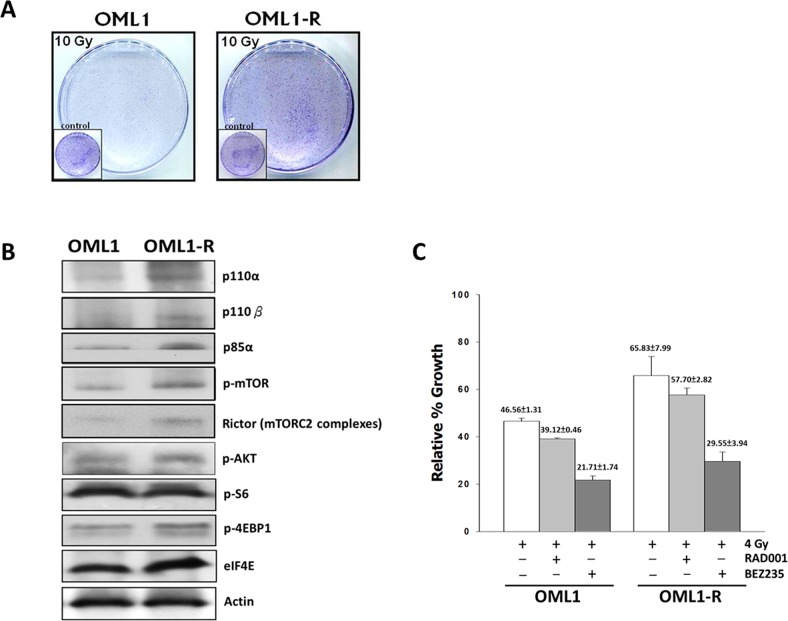
The dual PI3K/mTOR inhibitor reduces radiation survival of OML1-R and parental cells **(A)** Clonogenic assay in radioresistant OML1-R and parental OML1 cells using a single 10 Gy IR. Cells were allowed to recover for 14 days and then stained with 0.4% crystal violet. **(B)** Expression of AKT and mTOR signaling pathway molecules in OML1-R and OML1 cells. Cells were lysed and processed for western blot analysis. **(C)** The radiosensitizing effect of an mTORC1 inhibitor and BEZ235, a dual PI3K/mTOR inhibitor, in both cell lines. Cells were exposed to IR (4 Gy) with and without RAD001 (300 nM) or BEZ235 (100 nM). The colonies were imaged at 14 days. The number of colonies in each well was counted. Data represent the mean ± SD of three independent experiments performed in triplicate. SD = standard deviation.

To confirm the radioresistant phenotype of the OML1-R cell line, we determined the plating efficiency of the parental OML1 and the radioresistant OML1-R subline cells that were cultured after a high-dose fractionated IR exposure (10 Gy), and then examined using the clonogenic survival assay. OML1-R cells demonstrated significantly higher levels of clonal survival after IR in comparison with that of the parental cells (Figure 1A). We also analyzed the expression profiles of AKT/mTOR signaling pathway-related proteins; both p110 and p85 PI3K showed high expression levels in OML1-R cells. Additionally, phospho-AKT and mTOR downstream effectors, phospho-4EBP1 and eIF4E, displayed significantly higher expression levels in OML1-R cells (Figure 1B). Next, we determined that dual PI3K/mTOR inhibition with 100 nM BEZ235 in combination with IR dramatically inhibited the proliferation of OML1 and OML1-R cells compared to that by the mTORC1 inhibitor RAD001 with IR, or IR alone(Figure 1C). Thus, our findings suggest that the PI3K/Akt/mTOR signaling pathway is actively involved in OSCC radioresistance and that disruption of the PI3K/AKT/mTOR signaling pathway using the dual PI3K/mTOR inhibitor sensitizes cells to RT and overcomes OSCC radioresistance.

### Inhibiting the PI3K/AKT/mTOR signaling pathway enhances radiosensitization in OSCC cell lines and patient-derived cells

**Figure 2:**
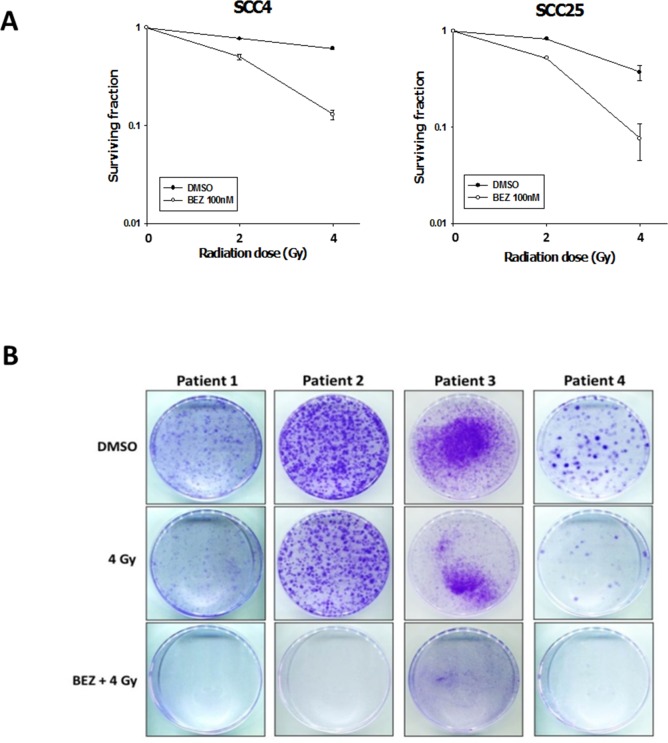
The dual PI3K/mTOR inhibitor enhances the radiosensitization of OSCC cells and patient-derived cells Effect of BEZ235 and IR on OSCC colony formation. **(A)** SCC4 and SCC25 cells were treated with AZD2014, IR, or the two treatments in combination to then perform a colony formation assay. Data represent the mean ± SD of three independent experiments **(B)** Patient-derived cells were established from four individual patients with OSCC. Typical images of colony growth for the different treatments are shown. The clinical and pathologic characteristics of patients are listed in Table 1.

We investigated whether targeting the PI3K/AKT/mTOR signaling pathway could sensitize OSCC cells to IR and reduce IR-induced radioresistance. For this purpose, we treated OSCC (SCC4, SCC25) and OML1-R cell lines with NVP-BEZ235 (50 or 100 nM) for 14 days, followed by IR (0–4 Gy). The combination of NVP-BEZ235 and IR clearly reduced proliferation of OSCC and radioresistant OSCC cell lines (Figure 2A). To confirm the clinical effectiveness of the dual PI3K/mTOR inhibitor, we isolated and expanded four primary tumor cells from OSCC patients and then treated them with NVP-BEZ235 with and without IR. Four cell lines derived from human OSCCs had been demonstrated for their different radiosensitivity. Cells derived from patients who had lymph node metastasis (N+) or local recurrence after radiotherapy revealed a higher colony growth after exposure to 4 Gy of IR when compared with those cells derived from the Patient 1 and 4. Consistently, the combination treatment of BEZ235 and IR significantly reduced colony formation in all primary OSCC cells (Figure 2B and Table 1). Thus, inhibition of the PI3K/AKT/mTOR signaling pathway enhances radiosensitivity in OSCC cells by repressing their colony formation ability.

**Table 1:** Information of patients and corresponding patient-derived cell models

	Patient 1	Patient 2	Patient 3	Patient 4
**Age/sex**	71/M	72/M	61/M	63/M
**Tumor type**	Squamous cell carcinoma	Squamous cell carcinoma	Squamous cell carcinoma	Squamous cell carcinoma
**Tumor location**	Left upper lip	Left buccal	Left cheek	Right tongue
**Tumor differentiation**	moderately	Moderately to pooly	moderately	Moderately to pooly
**Image staging**	cT2N0M0	cT3N1M0	rcT4aN0	cT4aN0M0
**Pathological Staging**	pT3N0	pT2N1	rpT4aN0	pT4aN0
**Recurrence**	No	No	Yes	No
**Patient has received radiation therapy**	No	No	Once	Once

### Combining a PI3K/mTOR signaling pathway inhibitor with radiation therapy is an effective anticancer strategy

**Figure 3:**
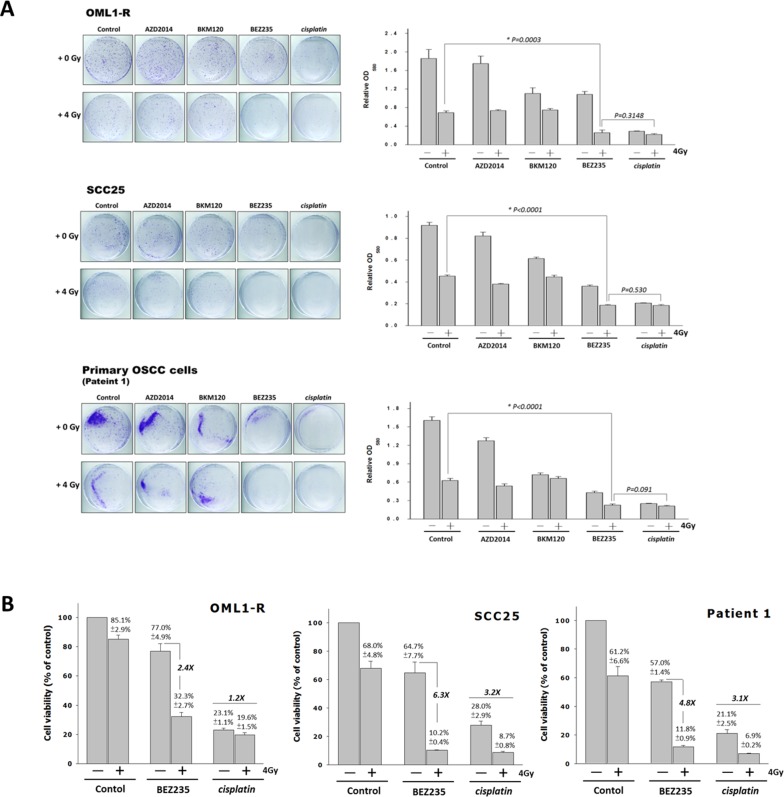
Comparison of the radiosensitizing effects of PI3K/mTOR inhibitors and Cisplatin in OSCC cells **(A)** SCC25, OML1-R, and patient-derived cells were treated with a dual PI3K/mTOR inhibitor, Cisplatin, and a single inhibitor (AZD2014 or BKM120) with or without IR for performing colony formation assays. Typical images of colony growth for the different treatments are shown. Quantitative analysis of individual colonies, stained cells were lysed and measured by a spectrophotometer, and the relative number of cells was expressed as OD580. Data were expressed as means ± SD (*n*=3). **(B)** Cells were treated with BEZ235 (100nM), cisplatin (0.5 mg/mL), and/or IR for 7 days. Cell viability was then analyzed by using MTT method. Values represent averages of three experiments, and bars indicate means ± SD. Graph shows fold change: BEZ235 combined with IR versus BEZ235 alone, and cisplatin combined with IR versus cisplatin alone.

Cisplatin has demonstrated radiosensitizing abilities of platinum-based compounds for the control of locoregional diseases in locally advanced head and neck cancers [3]; thus, we evaluated and compared the effects of the dual PI3K/mTOR inhibitor BEZ235, the pan-PI3K inhibitor BKM120, the mTORC1/mTORC2 inhibitor AZD2014, and Cisplatin with and without IR on SCC25, OML1-R, and primary OSCC cells. The combination treatment of BEZ235 and 4-Gy IR consistently showed a significant reduction in colony formation compared with that by each single PI3K or mTOR inhibitor (BKM120 or AZD2014) with or without IR. However, no significant differences were seen after cisplatin plus IR (Figure 3A) treatment in these three cell lines. In addition, we also found that AZD2014 was more effective than BKM120 in reducing clonogenic survival of SCC25 and primary OSCC cells.

In order to further confirm the PI3K/mTOR inhibitor to enhance IR-induced cytotoxicity more effectively, we examined cytotoxic effects of IR plus BEZ235 or cisplatin on cancer cells growth. We found that treatment of BEZ235 alone for oral cancer cell lines showed a 57%–77% survival rate, when combined with RT, significantly viability was reduced to 21%-32% in all cells. BEZ235 in combination with IR increased cell growth inhibition rates of 2.4 to 6.3 fold when compared with BEZ235 alone (Figure 3B). On the other hand, the addition of cisplatin obviously decreased the cell viability of OML1-R, SCC25 cells, and primary OSCC cells by 23.1%, 28.0% and 21.1%, and significantly reduced cell growth by 19.6%, 8.7% and 6.9% when combined with IR, respectively. When compared with cisplatin alone, combined IR increased a maximal 3-fold cell growth inhibition.

The above results showed that BEZ235 had a lower toxicity than cisplatin. More notably, when the cell lines were exposed to BEZ235 in conjunction with IR, cytotoxicity was obviously augmented. The tumor-killing effects of radiation were enhanced by adding BEZ235 in both radiosensitive and radioresistant OSCC cells. Our results suggested that inhibiting the PI3K/AKT/mTOR signaling pathway acts additively with IR, decreases proliferation of OSCC cells, and re-sensitizes resistant cells to IR obviously.

### Inhibition of the PI3K/AKT/mTOR signaling pathway sensitizes OSCCs to radiation by down-regulating the AKT/mTOR axis and inducing autophagic activity

**Figure 4:**
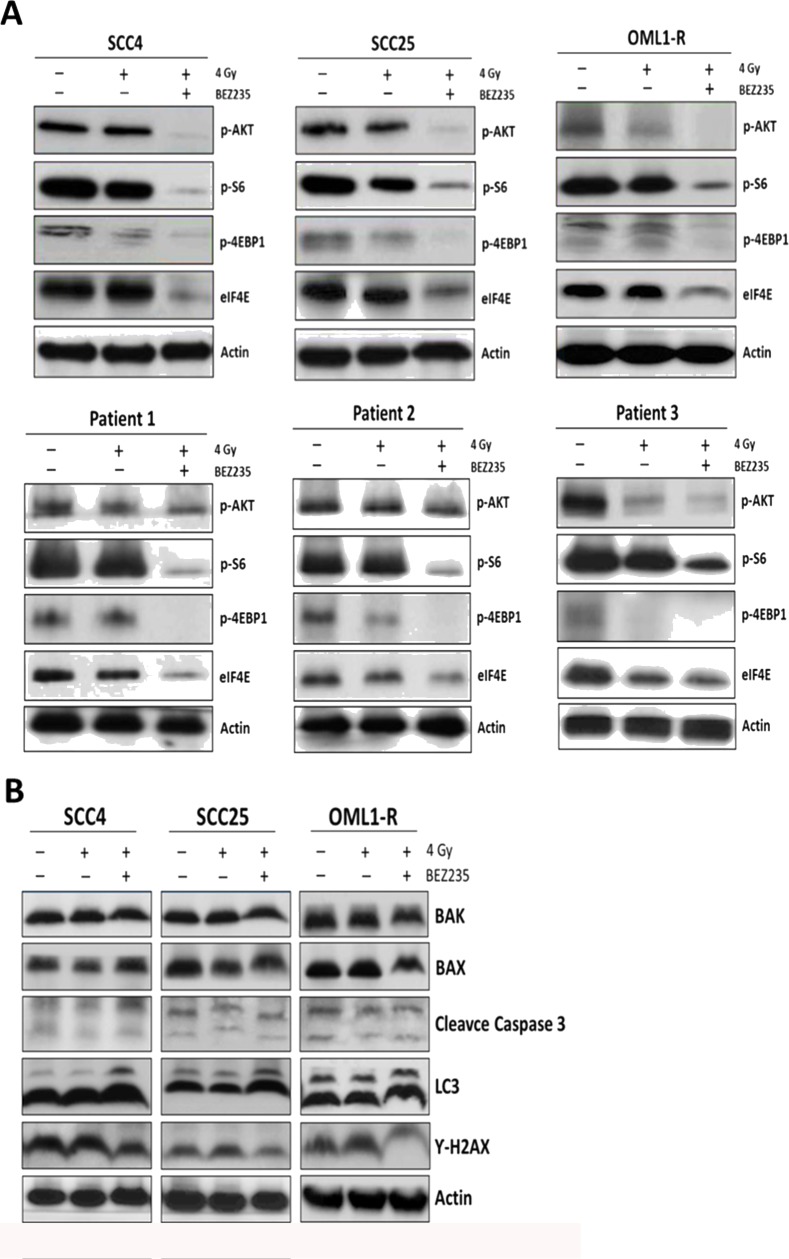
The dual PI3K/mTOR inhibitor sensitized OSCC cells to radiation, which led to reduced clonogenic survival by inhibiting AKT/mTOR signaling and inducing autophagy Cells were treated with IR alone or in combination with BEZ235. AKT/mTOR signaling pathway- **(A)** apoptosis-, autophagy-, and DNA damage pathway-related proteins **(B)** were analyzed by western blotting.

To investigate the involvement of the AKT/mTOR signaling pathway in the radiosensitization conferred by dual inhibition of the PI3K/mTOR signaling pathway, cells were treated with BEZ235 and/or exposed to 4 Gy of IR. Western blotting analyses revealed that treatment with BEZ235 diminished expression levels of phosphorylated AKT (phospho-AKT) in all OSCC cells. Consistently, we found that BEZ235-treated OSCC cells exposed to IR showed reduced levels of mTOR effectors including S6 kinase, 4EBP1, and eIF4E (Figure 4A). Therefore, inhibition of the PI3K/mTOR signaling pathway efficiently attenuated phosphorylation of AKT and mTOR signaling cascades, concomitantly blocking proliferation of OSCC cell lines, primary OSCCs, and radioresistant cells. We further examined whether cell death pathways (apoptosis and autophagy) were associated with OSCCs radiosensitivity after combination treatment of BEZ235 with IR. LC3 expression levels were increased after the combination treatment of BEZ235 and IR; however, neither apoptosis markers (Caspase 3, Bax, Bak) nor γ-H2AX, a sensitive marker of DNA damage response, showed significant differences after combination treatment (Figure 4B). Thus, these findings indicate that BEZ235 may be effective as a radiosensitizing agent against OSCC cell growth through enhanced inhibition of the AKT/mTORC axis and induction of autophagy activity.

### Inhibition of the PI3K/AKT/mTOR signaling pathway promotes G1 phase arrest by altering cell cycle-related proteins

**Figure 5:**
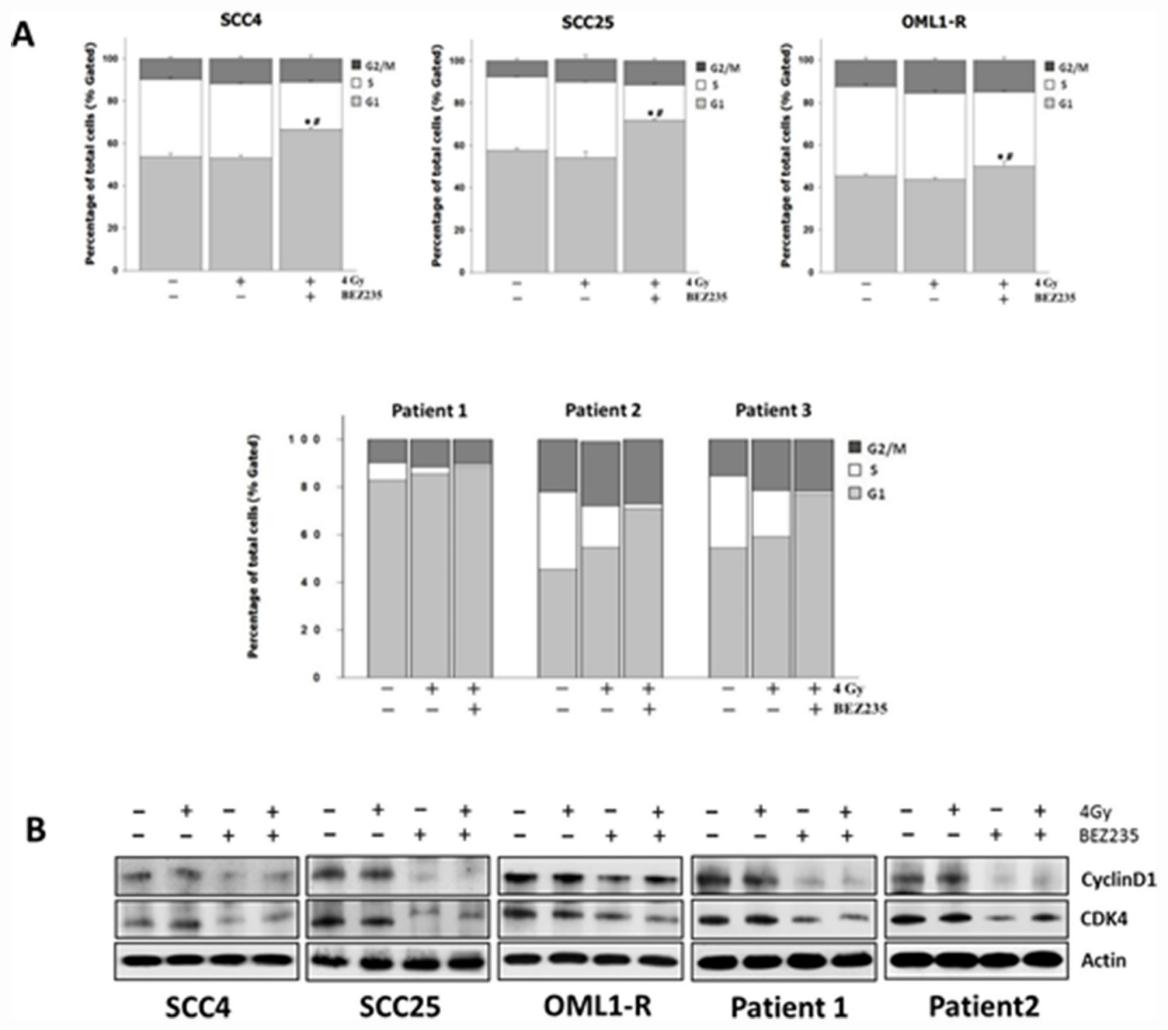
Combination treatment with the dual PI3K/mTOR inhibitor and IR causes G1 cell cycle arrest in OSCC cells by regulating cell cycle-related proteins **(A)** Cells were treated with BEZ235 with or without IR for 48 h. The cell cycle distributions were then evaluated. Data are representative of three independent experiments. **(B)** Expression of Cyclin D1 and CDK4 cell-cycle-related proteins in OSCC cells as determined by western blotting.

The PI3K/AKT/mTOR signaling pathway has been shown to play a distinct role in the regulation of the cell cycle [13]. Moreover, radiosensitization due to inhibition of the PI3K/AKT/mTOR signaling pathway has been shown to cause cell cycle arrest [14, 15]. Based on these findings, we demonstrated that BEZ235 significantly increased the proportion of OSCC cells (SCC4 and SCC25) and IR-resistant cells (OML1-R) in the G1 phase. Similar results were observed for three primary OSCC cell lines, which had a higher ratio of cells in the G1 phase after treatment with BEZ235. Additionally, the proportion of G2/M phase cells increased in IR-treated cells, whereas cells in the S phase decreased, indicating a G1/G2/M block (Figure 5A). In order to understand the molecular basis of the BEZ235-induced G1 arrest in cell growth inhibition, we investigated the probability of changes in expression levels of diverse regulators of the cell cycle, particularly those associated with G1 checkpoints such as cyclin D1, and CDK4 [16]. In the presence of BEZ235, cyclin D1 and CDK4 were significantly decreased; however, there were no obvious changes in the expression levels of these molecules in neither control nor IR-treated cells (Figure 5B). In agreement with the cell-cycle distribution analysis, inhibition of the PI3K/mTOR signaling pathway significantly reduced the expression of cyclin D1 and CDK4, causing cell cycle G1 arrest, thus contributing to the growth-inhibitory effects in all OSCC cells.

### Combination of PI3K/AKT/mTOR pathway inhibitors and RT enhanced growth inhibition in an *in vivo* tumor model

**Figure 6:**
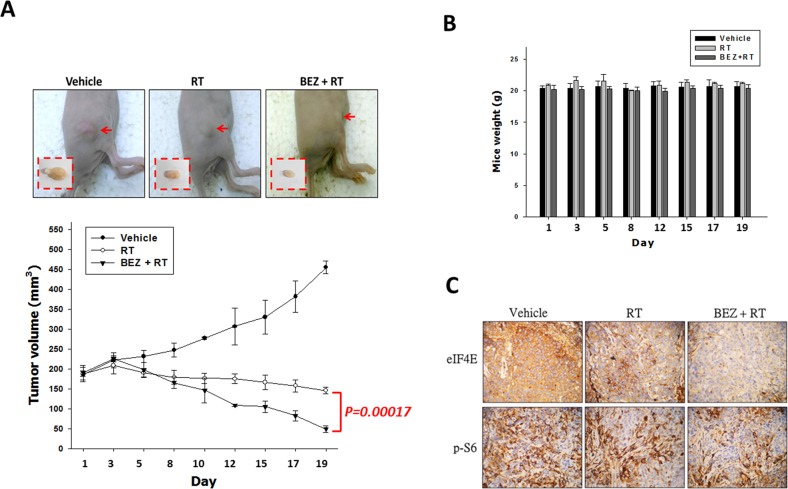
Treatment with a dual PI3K/mTOR inhibitor in combination with IR inhibits tumor growth of OML1-R xenografted tumor After subcutaneous injection of OML1-R oral cancer cells, athymic nude mice were treated with vehicle, IR alone (4 Gy), or with a combination of BEZ235 and IR. **(A)** As represented on the graph, tumor growth measurements were followed from 1 to 21 days as tumor volume. Tumors were measured after animals were sacrificed. **(B)** Body weight assessment after treatments. **(C)** Immunohistochemical analysis of phospho-S6 and eIF4E in xenograft tumors.

Next, we tested the role of the dual PI3K/mTOR inhibitor in tumor growth and radiosensitivity of an OML1-R xenografted mouse model. Mice bearing subcutaneous OML1-R tumors were randomized into three groups to receive: 1) IR alone (4 Gy fraction, thrice each week), 2) IR combined with BEZ235 (25 mg/kg BEZ235 twice weekly p.o.) or 3) sham treatment. IR treatment enhanced the suppression of OML1-R tumor growth compared to that by the control. IR in combination with BEZ235 significantly suppressed the growth of xenografted tumors to a greater extent than IR alone (*p* = 0.00017) (Figure 6A). The combination treatment did not significantly reduce mouse body weight (Figure 6B). To further confirm the biological significance of PI3K/mTOR inhibition *in vivo*, we examined the expression profile of mTOR signaling pathway-related molecules in tissue specimens of OML1-R xenografted tumors, three weeks after treatment. Immunohistochemistry analysis revealed a significant reduction in phospho-S6 and eIF4E levels in tumor-bearing radioresistant OML1-R mice treated with BEZ235 compared with that by either IR or control treatments (Figure 6C).

## DISCUSSION

Although RT is a powerful tool to treat oral cancer, resistance of tumor cells to this treatment remains a serious concern. Therefore, understanding the molecular mechanisms involved in the sensitivity of tumors to IR that lead to the acquisition of radioresistance and the identification of targets for its therapeutic use are important to improve RT and overcome radioresistance. The PI3K/AKT/mTOR signaling pathway has been extensively studied and demonstrated to be critical for RT resistance in various cancer types [17–19]. Although prior studies observed radiosensitization in response to treatment with dual PI3K/mTOR inhibitors (in other cancer cell lines both *in vitro* and *in vivo*) [20, 21], an *in vitro* condition does not truly represent clinical scenarios. Combining dual PI3K/mTOR inhibitors and IR treatment exhibits a synergistic anticancer effect on OSCC; however, this has only been partially explored and remains largely unknown. In this study, we used a radioresistant cell line and patient-derived OSCC cells to improve the understanding of radioresistance of oral cancer cells and the role of the PI3K/AKT/mTOR signaling pathway in radiosensitization.

The PI3K/AKT/mTOR signaling pathway is one of the most frequently dysregulated signaling cascades in human malignancies [13, 22] and has been known to play a pivotal role in the initiation and progression of malignancies, enhancement of cell survival, proliferation, and cellular metabolism. Moreover, this pathway has been implicated in radioresistance [17–19]. Drugs that target PI3K, or mTOR have been extensively developed and are being tested in clinical trials demonstrated that combined with radiation improved the anticancer effect. However, signaling events elicited by PI-3K and mTOR are complex and although overlapping, have non-identical functions that regulate cell survival and therapeutic resistance. We found higher expression levels of PI3K/AKT/mTOR-related proteins in radioresistant OSCC cell lines compared with that in the parental cell lines, suggesting that the PI3K/AKT/mTOR signaling pathway contributes to OSCC radioresistance. Numerous small molecular drugs targeting individual PI3K, AKT, or mTOR signaling proteins or dual blockade of PI3K and mTOR have been reported to induce a radiosensitizing effect. However, the key molecular acts in this pathway contributing to sensitize cells to IR still not clarified. We compared the inhibitory effects of these drugs and found that combining PI3K/AKT/mTOR inhibition with IR resulted in decreased survival of IR-resistant cells, patient-derived OSCC cells, and other OSCC cell lines. However, an mTORC1 inhibitor or PI3K inhibitor with or without IR could not repress tumor cell growth.

These data are consistent with previous our findings [23], Everolimus (RAD001) allosterically inhibits only mTORC1 but not mTORC2 causing feedback activation of AKT signaling due to not directly inhibit mTORC2 function, which can attenuate their antitumor activity. Conversely, dual mTORC1/mTORC2 inhibition increased the IR-induced inhibition of survival in both OSCC cell lines and patient-derived cells. Nevertheless, AZD2014 treatment did not affect radioresistant cells; results consistent with a previous study [12, 23]. BKM120 is a highly specific, orally available pan-Class I PI3K inhibitor that exhibits potent antiproliferative and synergistic radiosensitization in tumor cell lines [24, 25]. Our results demonstrated that NVP-BKM120 did not significantly enhance cell killing after IR exposure. Importantly, the combination of dual PI3K/AKT/mTOR inhibitors with IR significantly enhanced IR-induced regression of OSCC cells as efficiently as Cisplatin with IR treatment, indicating that dual PI3K/mTOR inhibition is a promising therapy that can potentially enhance the tumor response to radiation therapy in the clinic. These results provide a strong rationale for dual targeting of PI3K and mTOR activity to completely overcome radioresistance in OSCC and enhance radiosensitivity of OSCC cells, resulting in cell growth inhibition.

The PI3K/AKT/mTOR axis plays a crucial role in the regulation of cell growth migration, survival, and protein synthesis. Specifically, mTOR is a key regulator in mRNA translation and cell cycle progression via regulation of p70S6K and 4EBP1/eIF4E cascades [26, 27]. Moreover, mTOR signaling is often found activated and plays an important role in development of OSCC. It has been previously shown that inhibition of S6 phosphorylation via blocking of mTOR effectively suppressed, but did not completely prevent 4EBP1/eIF4E cascades, which may limit the utility of this therapeutic drug. Remarkably, although treatment with a dual PI3K/mTOR inhibitor led to efficient AKT inhibition and complete inhibition of both p70S6K and 4EBP1/eIF4E signaling, it showed a marked inhibitory effect on OSCC growth. These results support the finding that the anti-tumor effects of OSCC are required for effective inhibition of both PI3K and mTOR signaling pathways.

The S6K1 and 4EBP1/eIF4E signaling pathways have been shown to be critical for mTOR-mediated cell cycle progression. Both S6K1 and 4EBP1/eIF4E have been implicated in rapamycin-induced retardation of G1 cell cycle progression [28]. The eukaryotic initiation factor 4E (eIF4E) plays an important role in mRNA translation by binding the 5′-cap structure of the mRNA and promoting the formation of the translation initiation complex and ribosome binding. Expression of eIF4E is commonly elevated in human and experimental cancers and has been associated with promoting angiogenesis and tumor growth. Elevated eIF4E expression levels selectively elevate the expression of the oncogenes, c-myc and cyclin D1, which are involved in cell-cycle processes [29]. The previous results strongly suggested that suppression of eIF4E is also important for complete G1 cell cycle arrest. To further assess the synergistic effects of these proteins in the all tested OSCC cells after IR or combined drug-IR treatment, we analyzed the cell cycle phase distributions. We found that inhibition of the PI3K/AKT/mTOR signaling pathway causes cell cycle arrest in the G1 phase. Additionally, expression levels of cyclin D1 and CDK4, regulators of the G1 phase, were significantly downregulated. It was also reported that inhibition of mTOR can cause G1 arrest in a manner that could be suppressed through 4EBP1 phosphorylation; however, mTOR inhibition is not sufficient to suppress eIF4E in most cells [12, 23, 30].

Although de-repression of eIF4E was observed in BEZ235-treated OSCC cells, the inhibition of the cyclin D1/CDK4 complex activity leads to G1 cell cycle progression. These data demonstrate synergistic effects of combining a dual PI3K/mTOR inhibitor with IR to repress both S6K and 4EBP1/eIF4E signaling pathways and induce G1 arrest by inhibiting cyclinD1 and CDK4 activities, resulting in increased sensitivity towards IR. These data suggest that eIF4E represents a logical therapeutic target to increase tumor cell radiosensitivity and overcome cancer radioresistance that may support potential clinical value of eIF4E-targeting strategies for oral cancer treatment. Additionally, we examined the effect of combination treatment with dual or single IR on the induction of apoptosis and autophagy. Treatment with BEZ235 in combination with IR caused a slight induction of autophagy, whereas it had no relevant effect in apoptosis of OSCC cells for any of the treatments. γ-H2AX formation has normally been associated with the induction of double-strand breaks after exposure to IR or other DNA-damaging agents [31], and has been previously shown to be associated with radiosensitization after mTOR inhibition [32]. However, we did not observe any notable differences in phosphorylation of γ-H2AX for any treatment.

Finally, we confirmed the safety and efficacy of dual PI3K/mTOR inhibition *in vivo*. BEZ235 displayed a statistically significant anti-tumor activity and synergy with IR against OML1-R xenografts and an attenuation effect of eIF4E and S6K phosphorylation. No significant weight loss or illness was observed during the study period, suggesting that this therapy may have a promising safety profile. Furthermore, combination treatment of BEZ235 and IR *in vivo* is a safe and effective treatment that offered a greater therapeutic gain than that of RT alone.

In summary, this study confirmed that PI3K/AKT/mTOR signaling pathway activation has a role in radioresistance of oral cancers. More notably, the present study declared that combining a novel agent of BEZ235 to inhibit the PI3K/mTOR signaling pathway is a potential therapeutic strategy for irradiating OSCC, including patient-derived cells, OSCC radioresistant cell lines, and xenografts. The radiosensitization efficiency of BEZ235 is achieved by impairing S6K1 and 4EBP1/eIF4E signaling pathways, inducing cell-cycle arrest, and inducing autophagic cell death that causes cell proliferation or cell growth inhibition. These data suggested that an inhibitor of the PI3K/AKT/mTOR signaling pathway appears to be a potential therapeutic drug candidate for restoring radiosensitivity in oral cancers that were intrinsically or consequently resistant to RT. More importantly, it demonstrates a potential to replace the standard cisplatin-based RT, which has serious secondary effects such as toxicity, and provide useful information to predict therapeutic efficacy of OSCC.

## MATERIALS AND METHODS

### Ethical considerations

This study was initiated after a formal approval of the institutional review board (IRB) of the Dalin Tzu Chi Hospital, Buddhist Tzu Chi Medical Foundation, Taiwan (approved number, B10302008). All experiments involved human samples were obtained from patients who had surgical tumor resection and signed an informed consent.

### Reagents and chemicals

BEZ235, RAD001, and BKM120 were provided by Novartis Pharmaceuticals Corporation (East Hanover, NJ, USA). AZD2014 was obtained from AstraZeneca (London, United Kingdom). Cisplatin was obtained from Fresenius-Kabi (Bad Homburg, Germany). Stock solution were prepared in DMSO, stored at −20°C, and diluted in fresh medium for each experiment.

### Cell lines and cell culture

SCC4 and SCC25, which were derived from a squamous cell carcinoma of the tongue, were purchased from the American Type Culture Collection (ATCC; Manassas, VA, USA), and cultured in DMEM/F12 containing 10% fetal bovine serum (FBS), 1% penicillin-streptomycin and 2 mM l-glutamine. OML1 and OML1-R (Radioresistant) cells were established by Hon-Yi Lin and Michael W.Y. Chan et al. [11] and maintained in RPMI1640 containing 10% FBS, 1% penicillin-streptomycin, and 2 mM l-glutamine. Cells were cultured at 37°C under a humidified atmosphere of 5% CO_2_ and 95% air.

### Tissue specimens and initial cell culture

The establishment of primary cultures was successfully attained from four tumor tissues of patients with oral squamous cell carcinoma (including the lips, buccal mucosa, cheek tissue, and tongue). The cells were maintained in keratinocyte growth media (KGM; ScienCell Research Laboratories, Carlsbad, CA) with 15% FBS. All patient-derived cells were isolated according to a protocol previously described [12].

### Clonogenic assay

The OSCC cell lines, radioresistant OSCC cells, and patient-derived cells were pre-treated with RAD001, BEZ235, BKM120, or cisplatin and irradiated at 0, 2, or 4 Gy. After 14 days, colonies (defined as groups of > 50 cells) were stained with 0.05% crystal violet and counted. Pictures were acquired using a digital camera to count the colonies.

### Cell cycle profiling

The effects on the cell cycle in OSCC cell lines, radioresistant OSCC cells, and patient-derived cells exposed to BEZ235 (100 nM) and/or IR for 48 h were assessed with the NucleoCounter NC-3000 (ChemoMetec, Allerød, Denmark), which is based on the analysis of DAPI-stained cells.

### Western blotting

After treatment, cells were harvested and lysed, and protein concentrations were measured using the Bio-Rad protein assay kit (Bio-Rad, Richmond, CA, USA). Fifty micrograms of protein from each sample were separated by SDS-PAGE and transferred to a PVDF membrane (Millipore, Billerica, MA, USA). After blocking with non-fat dry milk for 1 h, membranes were incubated with the corresponding primary antibodies [PI3K p110α, PI3K p110β, PI3K p85β, phospho-mTOR (Ser2448), Rictor, phospho-AKT (Ser473), phospho-S6 (Ser235/236), phospho-4EBP1(Ser65), eIF4E, BAK, BAX, cleaved Caspase3, LC3, CyclinD1, CDK4, and γ-H2AX] and secondary antibodies for 1 h. All antibodies used in this study were obtained from Cell Signaling Technology (Beverly, MA, USA). Reactive bands were visualized using a chemiluminescence (ECL) detection kit (Millipore).

### *In vivo* radioresistance of mouse xenografts

Radioresistance of OML1-R transplanted xenografts in nude mice was established according to an already established method [11]. Male 3-week-old athymic nude mice (BALB/cAnN.Cg-Foxn1nu/CrlNarl) were obtained from the National Laboratory Animal Center, Taiwan. OML1-R cells (2 × 10^6^ cells) were injected subcutaneously into the flank of each mouse. When tumors reached a size of approximately 100 mm^3^, mice were randomized into three groups: Vehicle control, RT, and BEZ235+RT. BEZ235 was formulated in DMSO and intraperitoneally injected at a dose of 25 mg/kg three times in one week. Mice in IR groups were irradiated with a single dose of 4 Gy fractions two times in one week. Tumor volume was monitored and presented as the tumor volume growth ratio (final volume/initial volume).

### Immunohistochemistry

Tumor specimens from mice were fixed with 10% formaldehyde and embedded in paraffin. Five micrometer sections were cut, deparaffinized, and stained using a mouse monoclonal antibody for phospho-S6 (Ser235/236) and eIF4E (1:50, Cell Signaling Technology).

### Statistical analysis

All data were presented as the mean ± standard deviation. Significance levels were calculated by using Student's t-test, and p-values of less than 0.05 were considered statistically significant.

## Abbreviations

RT: radiation therapy
OSCC: oral squamous cell carcinoma
IR: ionizing radiation
